# Quantitative Analysis of Relationship Between Hypokinetic Dysarthria and the Freezing of Gait in Parkinson’s Disease

**DOI:** 10.1007/s12559-018-9575-8

**Published:** 2018-06-27

**Authors:** Jiri Mekyska, Zoltan Galaz, Tomas Kiska, Vojtech Zvoncak, Jan Mucha, Zdenek Smekal, Ilona Eliasova, Milena Kostalova, Martina Mrackova, Dagmar Fiedorova, Marcos Faundez-Zanuy, Jordi Solé-Casals, Pedro Gomez-Vilda, Irena Rektorova

**Affiliations:** 1 Department of Telecommunications, Brno University of Technology, Technicka 10, 61600 Brno, Czech Republic; 2 First Department of Neurology, St. Anne’s University Hospital, Pekarska 53, 656 91 Brno, Czech Republic; 3 Applied Neuroscience Research Group, Central European Institute of Technology, Masaryk University, Kamenice 5, 62500 Brno, Czech Republic; 4 Department of Neurology, Faculty Hospital and Masaryk University, Jihlavska 20, 63900 Brno, Czech Republic; 5 Escola Superior Politecnica, Tecnocampus, Avda. Ernest Lluch 32, 08302 Mataro, Barcelona Spain; 6 Data and Signal Processing Research Group, University of Vic – Central University of Catalonia, Perot Rocaguinarda 17, 08500 Vic, Catalonia Spain; 7 Neuromorphic Processing Laboratory (NeuVox Lab), Center for Biomedical Technology, Universidad Politécnica de Madrid Campus de Montegancedo, s/n, 28223, Pozuelo de Alarcón, Madrid Spain

**Keywords:** Parkinson’s disease, Hypokinetic dysarthria, Freezing of gait, Acoustic analysis, Quantitative analysis

## Abstract

Hypokinetic dysarthria (HD) and freezing of gait (FOG) are both axial symptoms that occur in patients with Parkinson’s disease (PD). It is assumed they have some common pathophysiological mechanisms and therefore that speech disorders in PD can predict FOG deficits within the horizon of some years. The aim of this study is to employ a complex quantitative analysis of the phonation, articulation and prosody in PD patients in order to identify the relationship between HD and FOG, and establish a mathematical model that would predict FOG deficits using acoustic analysis at baseline. We enrolled 75 PD patients who were assessed by 6 clinical scales including the Freezing of Gait Questionnaire (FOG–Q). We subsequently extracted 19 acoustic measures quantifying speech disorders in the fields of phonation, articulation and prosody. To identify the relationship between HD and FOG, we performed a partial correlation analysis. Finally, based on the selected acoustic measures, we trained regression models to predict the change in FOG during a 2-year follow-up. We identified significant correlations between FOG–Q scores and the acoustic measures based on formant frequencies (quantifying the movement of the tongue and jaw) and speech rate. Using the regression models, we were able to predict a change in particular FOG–Q scores with an error of between 7.4 and 17.0 %. This study is suggesting that FOG in patients with PD is mainly linked to improper articulation, a disturbed speech rate and to intelligibility. We have also proved that the acoustic analysis of HD at the baseline can be used as a predictor of the FOG deficit during 2 years of follow-up. This knowledge enables researchers to introduce new cognitive systems that predict gait difficulties in PD patients.

## Introduction

The primary motor symptoms of Parkinson’s disease (PD) comprise tremor at rest, muscular rigidity, progressive bradykinesia and postural instability [38, 40]. Patients with PD also develop additional axial motor symptoms [40] that consist of dysarthria, dysphagia, gait freezing, etc. A variety of non-motor symptoms such as sleep disturbances, depression, cognitive impairment/dementia, sensory problems and other symptoms can also be present [10, 17, 37]. PD is an incurable disorder affecting the activities of daily living and quality of life.

According to the previous studies [57], up to 90*%* of PD patients suffer from hypokinetic dysarthria (HD) [16]. HD is a distinctive motor speech disorder [9] that is manifested in the area of phonation (insufficient speech breath support, reduction in phonation time, a harsh breathy voice quality, etc.) [28, 30, 36, 70, 80], articulation (reduced variability of the articulatory organs’ mobility, imprecise consonant articulation, etc.) [1, 11, 12, 67] and speech prosody (monopitch, monoloudness and speech rate/pausing abnormalities) [20, 22, 29, 46, 63]. HD leads to serious complications in daily vocal-prosodic communication of patients with PD [34, 56].

Freezing of gait (FOG) is also a common and disabling feature in people with PD that is characterized by sudden and transient interruptions to walking; FOG frequently occurs when initiating walking, turning or facing an obstacle or narrow path [52]. FOG significantly reduces the patient’s mobility, independence and quality of life. It has been diagnosed in approximately half of the patients with PD and is more likely to occur in advanced stages of the disease [5]. According to the previous studies [23, 26, 44], patients suffering from FOG experience problems in controlling and modulating their gait, especially during its initiation and changes in direction or speed. Gait disorders associated with FOG in PD (reduction of velocity and step length compensated by increased cadence and stride-to-stride variability, etc.) [23, 26, 50] can lead to poor locomotion, postural instability and eventually to serious fall-related injuries [33, 62]. Despite the fact that FOG is a very problematic aspect occurring in most patients with PD [27], the exact pathophysiological mechanism underlying FOG in PD remains unexplained [64, 73, 75].

FOG and HD are both disabling axial symptoms of PD that do not sufficiently respond to dopaminergic medication [4, 9, 29, 55, 71]. Although it is expected that HD and FOG have some common pathomechanisms, not much research exists addressing a relationship between FOG and HD in PD. Giladi et al. ([25]) observed a strong association between FOG and speech abnormalities, both assessed by the Unified Parkinson’s Disease Rating Scale, part III: Motor examination (UPDRS III) [19]. However, due to the very limited ability of item 18 of this scale to sufficiently describe multidimensional HD, the authors simply conclude there might be some common pathophysiological mechanisms that should be studied further.

Consequently, Bartels et al. ([5]) studied a relationship between FOG (quantified by FOG frequency, which is based on a performance of a 130-m walk) and other clinical features of PD (evaluated by UPDRS, including speech) in 19 patients who were assessed in their OFF (prior to levodopa intake) and ON (after the levodopa dosage use) state. They reported that the FOG frequency was not correlated with other parkinsonian features in the OFF state and it was related to speech and writing only in the ON state.

Two years later, A. M. Goberman ([29]) published a work that deals with the correlation analysis between 16 acoustic speech measures (quantifying phonation, articulation and prosody) and non-speech motor performance (assessed by UPDRS) in nine PD patients. Three significant and positive correlations were identified between gait deficits and these acoustic parameters: the standard deviation of fundamental frequency, percent pause time calculated from monologue, and finally percent pause time calculated from a reading task.

Moreau et al. ([49]) were interested in the relationship between oral festination (quantified by parameters based on diadochokinetic rate), and gait festination and FOG separately. They enrolled 40 PD patients for this study (17 presented both gait festination and FOG, 5 presented gait festination alone, 9 presented FOG alone and 9 did not present either FOG or festination) and observed that oral festination was associated more to the gait festination than to the severity of FOG.

Cantiniaux et al. ([13]) measured walking velocity, step length and walking cadence in 11 PD patients undergoing the deep brain stimulation of the subthalamic nucleus (STN-DBS) using an optoelectronic system. In addition, they calculated speech rate, net speech time and speech index of rhythmicity employing the acoustic analysis of speech. Based on the correlation analysis, they concluded that speech rate and walking velocity as well as net speech time and step length significantly correlate. Negative correlation was identified between speech index of rhythmicity and walking cadence. The authors conclude that similar fundamental hypokinetic impairment and probably a similar rhythmic factor affect the patients’ speech and gait in a similar way.

In 2014, Park et al. ([54]) performed correlation analysis among several FOG features (gait velocity, stride length and cadence), evaluated by the Gait and Falls Questionnaire (GFQ) and the Freezing of Gait Questionnaire (FOG–Q), and three speech parameters (initiation time, rate, dysfluency) in 18 PD patients (9 with FOG, 9 without FOG). They reported that the increase in gait velocity positively correlated with the decrease in the time delay of the speech initiation, the increase in the gait velocity and cadence positively correlated with the decrease in the number of repetitions per sentence (dysfluency), and finally, the increase in the stride length positively correlated with the increase in speech rate and decrease in the number of repetitions per sentence.

In 2016, McCaig et al. ([45]) analysed the effect of concurrent walking on speech production in 15 PD patients with hypophonia. More specifically, they analysed the effect of sitting, standing and three concurrent walking tasks on speech intensity and speech rate. They observed that concurrent walking produces a significant increase in speech intensity, relative to standing and sitting, while the same task has no effect on the speech rate. The faster the walking, the more intense the speech. Finally, they reported that the concurrent walking and talking produced significant reductions in walking speed.

Rektorova et al. [58] assessed whether the baseline acoustic parameters, alone or in combination with other motor and non-motor symptoms may predict change in cognitive status and cognitive decline during a 2-year follow-up. The speech index of rhythmicity predicted a cognitive status change with 73.2 % accuracy (sensitivity 87.1 %, specificity 30.0 %) while adding FOG in the multivariate model improved the accuracy by 4.8 %, thus suggesting that both HD and FOG parameters relate to cognitive impairment in PD.

Finally, Ricciardi et al. ([59]) were investigating the relationship between speech disturbances (assessed perceptively by the Italian version of the Dysarthria Profile, which is made of eight sub-sections: respiration, phonation, facial musculature, diadochokinesis, articulation, intelligibility, rate/prosody, eating and swallowing) and FOG (evaluated by UPDRS II and the New Freezing of Gait Questionnaire) in 43 PD patients. They discovered that patients with FOG or with Hoehn-Yahr $>$ 2 reported lower scores in the articulation, intelligibility and rate/prosody sub-sections. Moreover, based on the multiple regression analysis, they proved that the severity of FOG is associated with the rate/prosody score only. Therefore, they conclude that it is especially speech dysfluency which shares pathophysiological mechanisms with FOG.

To sum up, although the authors cited proved that HD and FOG have some common pathophysiological mechanisms, none of the works explored the relationships using a complex acoustic analysis quantifying all the main disorders of HD. In addition, most of the research used a very simple FOG assessment based on UPDRS scores. Finally, until now, no researchers have dealt with a possibility of FOG deficit prediction using acoustic analysis of HD at baseline. Considering these facts, in the frame of this study, we aim to:

1. Identify significant relationships among 14 acoustic measures (providing a complex description of HD) and FOG symptoms,
2. Explore which dimension of HD (phonation, articulation and prosody) is mostly associated with FOG and
3. Find a combination of acoustic measures that can predict an FOG deficit (expressed using the Freezing Of Gait Questionnaire—FOG–Q) [24] in the horizon of 2 years.

We expect the research conducted in this work will have a high impact on the field of Health 4.0, which is utilizing cognitive computing systems. The cognitive systems have already been advantageously applied in the field of neurodegenerative disorders analysis. For instance, Gomez-Vilda et al. ([30]) proposed a cognitive-based approach of PD diagnosis and assessment using several measures of vocal fold biomechanics. Arias-Vergara et al. ([3]) employed a multi-class support vector machine (SVM) and a cognitive-inspired classifier (based on neural networks) to discriminate between the speech of young people, PD patients, and age-matched adults. Finally, Lopez-de-Ipina et al. ([39]) proved that cognitive systems can be used for the diagnosis of Alzheimer’s disease (AD) as well. Moreover, cognitive systems assessing neurodegenerative diseases are not limited to acoustic speech analysis only. For example, Rosenblum and Luria ([61]) proposed a system that can be used to monitor cognitive deficits in PD and AD patients using an online handwriting analysis. The advantages and limitations of in-air trajectories analysis for the purpose of PD, AD and mild cognitive impairment (MCI) diagnosis were discussed by Alonso-Martinez et al. ([2]).

Based on the above-mentioned research, it is evident that the cognitive systems have their important place in the field of quantitative neurodegenerative diseases analysis. Therefore, a cognitive system that predicts an FOG deficit based on acoustic HD assessment would empower neurologists to remotely (using smart devices) and effectively monitor a patient’s state of health, individualize treatment or intervene if necessary (e.g. to prevent falls). However, in order to be able to propose this cognitive-inspired system, we need, as a first step, a deep knowledge of the pathophysiological mechanisms that, in consequence, could be emulated. This study provides a complex analysis of these mechanisms for the research community.

The remainder of this paper is organized as follows. Section “Patients and Methods” presents the cohort of patients, the quantitative HD analysis approach and statistical data processing methods. Section “Results” provides statistics and experimental results that are consequently discussed in “Discussion”. Finally, conclusions are given in “Conclusions”.

## Patients and Methods

### Study Participants

We enrolled 75 non-depressed patients with PD (48 males/27 females; mean age 67.40 $\pm $ 7.95 years) at the First Department of Neurology, St. Anne’s University Hospital in Brno, Czech Republic. After 2 years, 41 of these patients (27 males/14 females; mean age 67.34 $\pm $ 7.60 years) were re-examined. For clinical data, see Table 1. None of the patients had a disease affecting the central nervous system other than PD.

**Table 1:** Patients’ clinical data

Clin. data	Mean	std	Min	Median	Max
Session 1 (48 males/27 females)					
PD dur. (years)	7.48	4.15	1	7	21
UPDRS III	23.89	12.05	3	25	55
LED (mg)	997.26	554.05	0	870	2275
NMSS	35.60	20.58	2	33	94
RBDSQ	3.76	3.22	0	3	13
MMSE	27.97	2.49	16	29	30
ACE-R	87.11	7.98	60	88	99
BDI	10.51	6.08	0	9	27
FOG (Q3)	1.49	1.55	0	1	4
FOG (Q4)	1.09	1.30	0	1	4
FOG (Q5)	0.92	1.19	0	0	4
FOG (Q6)	0.75	1.03	0	0	4
FOG (total)	4.25	4.57	0	3	16
Session 2 (27 males/14 females)					
PD dur. (years)	9.68	4.69	4	9	24
UPDRS III	28.15	12.93	5	29	61
LED (mg)	1128.67	469.20	375	1070	2852
NMSS	55.54	33.72	2	57	138
RBDSQ	3.61	2.29	0	3	10
MMSE	28.02	2.08	22	29	30
ACE-R	84.88	9.68	51	87	97
BDI	10.76	5.12	2	10	25
FOG (Q3)	1.71	1.50	0	2	4
FOG (Q4)	1.22	1.31	0	1	4
FOG (Q5)	1.24	1.20	0	1	4
FOG (Q6)	1.05	1.16	0	1	4
FOG (total)	5.22	4.76	0	6	16

*LED* l-dopa equivalent daily dose (mg/day) [42]

The following questionnaires and scales were used to evaluate the clinical symptoms of PD: UPDRS, part III: Motor Examination [19], Non-Motor Symptom Scale (NMSS) [15], REM Sleep Behavioral Disorder Screening Questionnaire (RBDSQ) [68], Beck Depression Inventory (BDI) [43], Mini Mental State Examination (MMSE) [21] and Addenbrooke’s Cognitive Examination, revised (ACE-R) [7, 48]. Freezing of gait has been assessed using FOG–Q [24]. It is a six-item questionnaire (5-point scale: a score of 0 specifies absence of any symptoms; a score of 4 specifies the most severe stage of the disease) that can be theoretically split into two parts:

1. *General questions*
  Q1: During your worst state, do you walk: …
  Q2: Are your gait difficulties affecting your daily activities and independence?
2. *Quantitative description of freezing*
  Q3: Do you feel that your feet get glued to the floor while walking, making a turn or when trying to initiate walking (freezing)?
  Q4: How long is your longest freezing episode?
  Q5: How long is your typical start hesitation episode (freezing when initiating the first step)?
  Q6: How long is your typical turning hesitation (freezing when turning)?

Since we were more interested in a specific description of freezing, we statistically processed only Q3–Q6 and their sum (total score) (see Table 1).

All patients were examined on their regular dopaminergic medication (ON state) approximately 1 h after the l-dopa dose. The study was approved by the local ethics committee, and all the patients signed an informed consent form.

### Acoustic Voice and Speech Analysis

The speech acquisition protocol was derived from the standardized 3F Dysarthria Profile [41] and included 14 vocal tasks that were used to quantify the main HD disorders (see Table 2).

**Table 2:** List of the vocal tasks

Code	Vocal task	Description
TSK1	Monologue	Monologue without the interruption of a clinician. The participants were instructed to speak about their hobbies, family, etc.
TSK2	Expiration with closed lips	Sustained phonation of the consonant /m/ with closed lips as constantly and long as possible. Performed in one breath.
TSK3	Expiration with open lips	Sustained phonation of the vowel /i/ with open lips as constantly and long as possible. Performed in one breath.
TSK4	Sustained phonation	Sustained phonation of the vowel /a/ at a comfortable pitch and loudness. Performed in one breath and without any limitations in length.
TSK5	Diadochokinetic task	Rapid steady /pa/-/ta/-/ka/ syllable repetition as constantly and long as possible. Performed in one breath.
TSK6	Rhythmical units	Reading a text containing 4 rhymes of 16 words rhythmically.
TSK7	Basic intonation template	Short sentence reading containing 3 words and pronounced interrogatively.
TSK8	Basic intonation template	Short sentence reading containing 3 words (same as in TSK7) and pronounced imperatively.
TSK9	Basic intonation template	Short sentence reading containing 3 words (same as in TSK7) and pronounced declaratively.
TSK10	Reading paragraph	Reading a phonetically non-balanced text of 135 words.
TSK11	Reading with different emotions	Reading a sentence of 8 words neutrally.
TSK12	Reading with different emotions	Reading a sentence of 6 words angrily.
TSK13	Reading with different emotions	Reading a sentence of 9 words in a bored manner.
TSK14	Reading with different emotions	Reading a sentence of 5 words excitedly.

The vocal tasks were recorded using a large capsule cardioid microphone M-AUDIO Nova mounted to a boom arm RODE PSA1 and positioned at a distance of approximately 20 cm from the patient’s mouth. Signals were digitized by audio interface M-AUDIO Fast Track Pro using a sampling frequency $f_{\text {s}} = 48$ kHz and 16-bit resolution. The signals were parametrized using Praat [8] and specially developed Matlab functions [47] by a trained acoustic engineer without having seen the patient’s clinical data.

We calculated a set of acoustic measures (see Table 3) that is based on a recommendation we have given in our recent review on acoustic HD analysis [9]. This set contains features frequently used for the quantification of 14 specific HD disorders.

**Table 3:** Overview of acoustic features used for the quantification of specific HD disorders

HD dimension and specific disorder	Vocal tasks	Acoustic feature	Feature definition
Phonation			
Airflow insufficiency	Expiration with closed (TSK2) or opened (TSK3) lips	MPT	Maximum phonation time, aerodynamic efficiency of the vocal tract measured as the maximum duration of the sustained vowel/consonant.
Irregular pitch fluctuations	Sustained phonation (TSK4)	relF0SD	The standard deviation of fundamental frequency relative to its mean, variation in frequency of vocal fold vibration.
Microperturbations in frequency	Sustained phonation (TSK4)	jitter	Frequency perturbation, the extent of variation of the voice range. Jitter is defined as the variability of the F0 of speech from one cycle to the next. In this case it is implemented as the five-point period perturbation quotient.
Microperturbations in amplitude	Sustained phonation (TSK4)	shimmer	Amplitude perturbation, representing rough speech. Shimmer is defined as the sequence of maximum extent of the signal amplitude within each vocal cycle. In this case implemented as the five-point amplitude perturbation quotient.
Tremor of jaw	Sustained phonation (TSK4)	F1SD, F2SD	The standard deviation of the first (F1) and second (F2) formant. Formants are related to the resonances of the oro-naso-pharyngeal tract and are modified by position of tongue and jaw.
Increased noise	Sustained phonation (TSK4)	mean HNR	Harmonics-to-noise ratio, the amount of noise in the speech signal, mainly due to incomplete vocal fold closure. HNR is defined as the amplitude of noise relative to tonal components in speech.
Aperiodicity	Sustained phonation (TSK4)	DUV	Degree of unvoiced segments, the fraction of pitch frames marked as unvoiced.
Articulation			
Rigidity of tongue and jaw	Monologue (TSK1), rhythmical units (TSK6), basic intonation template (TSK7–9), reading paragraph (TSK10), reading with different emotions (TSK11–14)	F1IR, F2IR, F1SD, F2SD	Interpercentile range (range between 1st and 99th percentile) and standard deviation of the first (F1) and second (F2) formant. Formants are related to the resonances of the oro-naso-pharyngeal tract and are modified by position of tongue and jaw.
Slow alternating motion rate	Diadochokinetic task (TSK5)	DDK rate	Diadochokinetic rate, representing the number of syllable vocalizations per second.
Irregular alternating motion rate	Diadochokinetic task (TSK5)	DDK reg	Diadochokinetic regularity, defined as the standard deviation of distances between following syllables nuclei.
Prosody			
Monoloudness	Monologue (TSK1), rhythmical units (TSK6), basic intonation template (TSK7–9), reading paragraph (TSK10), reading with different emotions (TSK11–14)	relSEOSD	Speech loudness variation, defined as a standard deviation of intensity contour relative to its mean.
Monopitch		relF0SD	Pitch variation, defined as a standard deviation of F0 contour relative to its mean.
Inappropriate silences	Reading paragraph (TSK10)	SPIR	Number of speech inter-pauses per minute.
Unnatural speech rate	Basic intonation template (TSK7–9), reading paragraph (TSK10), reading with different emotions (TSK11–14)	TSR, NSR	If we consider total speech time (TST) as a duration of the whole speech, and net speech time (NST) as a duration of speech without pauses, then the total speech rate (TSR) is defined as a number of phones per TST, and the net speech rate (NSR) as a number of phones per NST.

### Statistical Analysis

We first assessed the strength of a relationship between the patients’ clinical data and the FOG–Q scores in both sessions. For this purpose, we used Pearson’s correlation with the significance level set to $p = 0.05$. With this approach, we aimed to identify those clinical measures (PD duration, UPDRS III, LED, NMSS, RBDSQ, MMSE, ACE-R, BDI) that are significantly correlated with the specific symptoms of gait freezing in PD.

Next, we assessed the strength of a relationship between voice disorders associated with HD and freezing of gait in PD. We computed Pearson’s (linear relation), Spearman’s (monotonic relation) and Kendall’s (monotonic relation) partial correlation coefficients (controlling for the effect of other clinical factors) between the acoustic measures and the values of FOG–Q with the significance level of correlation set to $p = 0.05$. Because both age and gender have effect on HD speech disorders ([3, 66]), during the calculation of partial correlations we controlled for the effect of these factors. In addition, we controlled for the effect of dopaminergic medication LED (mg/day), and other symptoms associated with PD as measured by UPDRS III, BDI, and ACE-R. As in the previous case, we aimed to identify those acoustic measures that significantly correlated with the specific symptoms of gait freezing in PD.

Finally, to evaluate the power of the speech features (in Session 1) in predicting the change of the severity of gait freezing in PD (ΔFOG–Q), we employed gradient boosting trees (using stratified 10-fold cross-validation with 100 repetitions) [6, 69]. Gradient boosting algorithms currently belong to the state-of-the-art in machine learning and are consistently used to win competitions on Kaggle (www.kaggle.com). The advantage of the gradient boosting trees in comparison with other regression techniques is its inherent robustness to outliers and ability to use one variable more than once, which enables the uncovering of complex interdependencies.

The performance of the trained models was measured by mean absolute error (MAE), root mean squared error (RMSE), estimation error rate (EER), sum of squared errors (SSE) and coefficient of determination (*R*^2^). The metrics are defined by following equations:

1 $$\begin{array}{@{}rcl@{}} \text{MAE}&=&\frac{1}{n}\sum\limits_{i = 1}^{n} \left|y_{i}-\hat{y_{i}}\right|, \end{array} $$

2 $$\begin{array}{@{}rcl@{}} \text{RMSE}&=&\sqrt{\frac{1}{n}\sum\limits_{i = 1}^{n} (y_{i}-\hat{y_{i}})^{2}}, \end{array} $$

3 $$\begin{array}{@{}rcl@{}} \text{EER}&=&\frac{1}{n \cdot r}\sum\limits_{i = 1}^{n} \left|y_{i}-\hat{y_{i}}\right| \cdot 100\,[\%], \end{array} $$

4 $$\begin{array}{@{}rcl@{}} \text{SSE}&=&\sum\limits_{i = 1}^{n} (y_{i}-\hat{y_{i}})^{2}, \end{array} $$

5 $$\begin{array}{@{}rcl@{}} R^{2}&=&1 - \frac{\text{SSE}}{{\sum}_{i = 1}^{n} (y_{i}-\bar{y})^{2}}, \end{array} $$

6 $$\begin{array}{@{}rcl@{}} \bar{y}&=&\frac{1}{n}\sum\limits_{i = 1}^{n} y_{i}, \end{array} $$

where *n* denotes the number of true/predicted values, $y_{i}$ and $\hat {y_{i}}$ represents the true and predicted values of the response variable, respectively, and *r* denotes the range of values of the given clinical rating scale present in the dataset.

MAE expresses the average error in absolute values. While the MAE gives the same weight to all errors, the RMSE penalizes variance as it gives errors with larger absolute values more weight than errors with smaller absolute values [14]. EER quantifies the estimation error of the model given the actual statistical properties of the dataset. SSE is a measure of the discrepancy between the data and values estimated by a model. Finally, $R^{2}$ expresses the proportion of the variance in the dependent variable that is explained by the variation in predictor. Based on the work of Chai and Draxler ([14]), it is always better to provide more evaluation metrics in order to get a complex overview of the model’s performance. Although we evaluated the model based mainly on the EER (which we think is easily interpretable for clinicians), we left the selection of the final evaluation metric to a reader.

## Results

The values of Pearson’s correlation coefficients among FOG–Q items and clinical scores can be found in Table 4. In both sessions, we identified significant correlations of all items with duration of PD and with UPDRS III (except Q5 in the first session). Contrary to session 2, l-dopa equivalent daily dose (LED) (mg/day) significantly correlated with all FOG–Q items in session 1. Similarly, just a few significant correlations were observed with the NMSS score in session 2 as compared to session 1. Generally, FOG–Q items correlated variably with RBDSQ; however, the Q3–6 total score correlated significantly with this questionnaire in both sessions. The scales assessing cognitive functions (MMSE and ACE-R) did not significantly correlate with FOG either in session 1 or in session 2. The BDI score did not correlate significantly with FOG–Q in session 1, while we can find significant correlations in session 2. Regarding the correlations between ${\Delta }\text {Q3--6 total score}$ and ${\Delta }$ of the clinical scores, we identified significant correlations with changes of LED and MMSE.

**Table 4:** Correlations among FOG–Q items and clinical data

FOG–Q	Q3	Q4	Q5	Q6	Total
Session 1					
PD dur. (years)	0.47**	0.35**	0.35**	0.39**	0.44**
UPDRS III	0.24*	0.24*	0.21	0.23*	0.25*
LED (mg)	0.36**	0.33**	0.37**	0.24*	0.37**
NMSS	0.45**	0.43**	0.39**	0.50**	0.49**
RBDSQ	0.25*	0.28*	0.14	0.28*	0.27*
MMSE	$-0.01$	$-0.07$	0.06	$-0.06$	$-0.02$
ACE-R	$-0.06$	$-0.15$	0.04	$-0.13$	$-0.08$
BDI	0.05	0.06	0.09	0.13	0.09
Session 2					
PD dur. (years)	0.41**	0.41**	0.38*	0.42**	0.44**
UPDRS III	0.36*	0.45**	0.35*	0.39*	0.42**
LED (mg)	0.28	0.03	0.17	0.15	0.18
NMSS	0.39*	0.30	0.26	0.36*	0.36*
RBDSQ	0.34*	0.28	0.38*	0.41**	0.38*
MMSE	$-0.26$	$-0.14$	$-0.14$	$-0.07$	$-0.17$
ACE-R	$-0.25$	$-0.18$	$-0.18$	$-0.13$	$-0.20$
BDI	0.36*	0.36*	0.38*	0.38*	0.40**
Δ (session 2–session 1)					
PD dur. (years)	$-0.22$	0.06	0.13	0.25	0.04
UPDRS III	0.03	0.17	0.17	0.10	0.16
LED (mg)	$-0.28$	$-0.33$	$-0.35$	$-0.18$	$-0.40$*
NMSS	0.20	0.04	0.20	0.42*	0.28
RBDSQ	0.09	0.24	0.06	0.24	0.21
MMSE	$-0.35^{*}$	$-0.26$	$-0.29$	$-0.12$	$-0.36$*
ACE-R	$-0.17$	$-0.25$	$-0.24$	$-0.06$	$-0.25$
BDI	$-0.26$	$-0.10$	$-0.06$	0.02	$-0.16$

*LED* l-dopa equivalent daily dose (mg/day) [42]; *p < 0.05, **p < 0.01

The partial correlations among acoustic features and FOG–Q items are reported in Table 5. For a better overview, we selected only those features that correlated significantly in all of Pearson’s, Spearman’s, and Kendall’s correlations. Regarding Q3 (assessing occurrence of freezing), this item correlated mainly with the interpercentile range of the first formant (calculated from 5 different vocal tasks), and with net speech rate (calculated from short read sentence). In Q4 (assessing the duration of the longest freezing episode), we identified two significant negative correlations, in both cases with the interpercentile range of the first formant. Following item Q5 (assessing the duration of the typical start hesitation), this correlated significantly with the interpercentile range of the first formant, and with the net speech rate. In the last item Q6 (assessing the duration of the typical turning hesitation), we observed significant correlations only with the parameters quantifying the speech rate. Finally, the Q3–6 total score correlated significantly with the first formant statistics and with the net speech rate.

**Table 5:** Significant partial correlations among acoustic features and FOG–Q (session 1) items

HD dimension	Specific disorder	Acoustic feature	*r* (P)	*p* (P)	*r* (S)	*p* (S)	*r* (K)	*p* (K)
Q3								
Prosody	Unnatural speech rate	NSR (TSK8)	0.4130	0.0039	0.3355	0.0211	0.2660	0.0440
Articulation	Rigidity of tongue and jaw	F1IR (TSK10)	$-0.3988$	0.0055	$-0.4424$	0.0018	$-0.3448$	0.0083
Articulation	Rigidity of tongue and jaw	F1IR (TSK9)	$-0.3391$	0.0197	$-0.3773$	0.0089	$-0.3251$	0.0131
Articulation	Rigidity of tongue and jaw	F1IR (TSK13)	$-0.3172$	0.0298	$-0.3931$	0.0063	$-0.2857$	0.0301
Articulation	Rigidity of tongue and jaw	F1IR (TSK14)	$-0.2957$	0.0436	$-0.3037$	0.0379	$-0.2660$	0.0440
Articulation	Rigidity of tongue and jaw	F1IR (TSK7)	$-0.2951$	0.0440	$-0.3530$	0.0149	$-0.3153$	0.0162
Q4								
Articulation	Rigidity of tongue and jaw	F1IR (TSK10)	$-0.4016$	0.0051	$-0.4322$	0.0024	$-0.3054$	0.0201
Articulation	Rigidity of tongue and jaw	F1IR (TSK9)	$-0.3506$	0.0157	$-0.3998$	0.0054	$-0.2857$	0.0301
Q5								
Articulation	Rigidity of tongue and jaw	F1IR (TSK14)	$-0.4668$	0.0009	$-0.4888$	0.0005	$-0.4089$	0.0015
Prosody	Unnatural speech rate	NSR (TSK8)	0.3559	0.0141	0.4153	0.0037	0.2709	0.0401
Articulation	Rigidity of tongue and jaw	F1IR (TSK10)	$-0.3550$	0.0143	$-0.3990$	0.0055	$-0.2808$	0.0332
Articulation	Rigidity of tongue and jaw	F1IR (TSK13)	$-0.2928$	0.0458	− 0.3637	0.0120	$-0.2611$	0.0482
Q6								
Prosody	Unnatural speech rate	TSR (TSK11)	0.3285	0.0241	0.3307	0.0232	0.4236	0.0010
Prosody	Unnatural speech rate	NSR (TSK8)	0.3281	0.0244	0.3326	0.0223	0.3054	0.0201
Prosody	Unnatural speech rate	NSR (TSK11)	0.3162	0.0303	0.3249	0.0259	0.3103	0.0181
Total								
Articulation	Rigidity of tongue and jaw	F1IR (TSK10)	$-0.4049$	0.0048	$-0.4474$	0.0016	$-0.3054 $	0.0201
Articulation	Rigidity of tongue and jaw	F1IR (TSK14)	− 0.3811	0.0082	$-0.3775$	0.0089	$-0.3153$	0.0162
Prosody	Unnatural speech rate	NSR (TSK8)	0.3639	0.0119	0.3770	0.0090	0.2857	0.0301

*P* Pearson’s correlation, *S* Spearman’s correlation, *K* Kendall’s correlation

Table 6 contains results related to the FOG severity change prediction in a 2-year horizon. When considering the three HD dimensions separately, we predicted the change of Q3 score with a 14.36 % error (*R*^2^ = 0.82) using seven articulatory features describing the rigidity of the tongue and jaw. The change of the Q4 score was predicted with the lowest error of 17.96 % (*R*^2^ = 0.69) using four prosodic features quantifying monopitch, monoloudness and speech rate. The formant-based articulatory features provided the best prediction of the Q5 score change (8.20 % error, $R^{2} = 0.80$). Finally, the prosodic features assessing speech rate, speech fluency and monopitch enabled us to predict Q6 change with 10.13 % error (*R*^2^ = 0.73) and the Q3–6 total score with 13.89 % error (*R*^2^ = 0.75). A combination of all the acoustic features mentioned in Table 3 improved predictions in all cases.

**Table 6:** FOG deficits prediction using gradient boosting trees

FOG–Q	MAE	RMSE	EER	SSE	$R^{2}$	No.	Selected features
Articulation							
Q3	0.59 ± 0.19	0.69 ± 0.20	14.36 ± 4.58	3.03 ± 1.81	0.82 ± 0.10	7	F1IR (TSK7), F1SD (TSK6, 9, 11, 12), F2IR (TSK11), F2SD (TSK11)
Q4	0.67 ± 0.19	0.79 ± 0.22	18.29 ± 5.24	3.92 ± 2.12	0.79 ± 0.69	3	F1IR (TSK11), F2IR (TSK10, 11)
Q5	0.38 ± 0.17	0.50 ± 0.24	8.20 ± 3.62	1.79 ± 1.72	0.80 ± 0.13	6	F2IR (TSK1, 7, 8, 9, 14), F2SD (TSK7)
Q6	0.54 ± 0.22	0.69 ± 0.29	12.47 ± 5.22	3.29 ± 2.50	0.91 ± 0.27	3	DDK rate (TSK5), F1SD (TSK8), F2IR (TSK11)
Total	1.47 ± 0.49	1.82 ± 0.58	14.96 ± 4.98	21.17 ± 13.36	0.77 ± 0.16	7	F1IR (TSK11, 12, 13), F1SD (TSK9, 11), F2IR (TSK11), F2SD (TSK6)
Phonation							
Q3	1.00 ± 0.31	1.22 ± 0.35	24.54 ± 7.61	9.41 ± 5.24	0.48 ± 0.30	3	jitter (TSK4), MPT (TSK2, 3)
Q4	0.97 ± 0.22	1.12 ± 0.24	26.64 ± 6.05	7.60 ± 3.18	0.10 ± 0.25	3	jitter (TSK4), MPT (TSK3), shimmer (TSK4)
Q5	0.54 ± 0.19	0.68 ± 0.26	11.61 ± 4.04	3.06 ± 2.31	0.55 ± 0.51	4	F2SD (TSK4), MPT (TSK3), relF0SD (TSK4), shimmer (TSK4)
Q6	0.49 ± 0.19	0.63 ± 0.24	11.47 ± 4.43	2.62 ± 1.98	0.58 ± 0.34	3	DUV (TSK4), mean HNR (TSK4), MPT (TSK3)
Total	2.19 ± 0.62	2.60 ± 0.65	22.36 ± 6.33	41.61 ± 20.38	0.47 ± 0.42	2	jitter (TSK4), mean HNR (TSK4)
Prosody							
Q3	0.62 ± 0.21	0.74 ± 0.23	15.29 ± 5.07	3.49 ± 2.23	0.79 ± 0.11	11	NSR (TSK7, 11, 12, 14), relF0SD (TSK10), relSEOSD (TSK1), TSR (TSK7, 9, 10, 11, 14)
Q4	0.65 ± 0.18	0.75 ± 0.20	17.96 ± 4.87	3.49 ± 2.04	0.69 ± 0.31	4	NSR (TSK12), relF0SD (TSK10), relSEOSD (TSK8), TSR (TSK7)
Q5	0.42 ± 0.19	0.54 ± 0.28	9.06 ± 4.15	2.16 ± 2.23	0.75 ± 0.50	11	NSR (TSK9, 11), relF0SD (TSK7), TSR (TSK13), relSEOSD (TSK6, 7, 8, 11, 12), TSR (TSK7, 14)
Q6	0.44 ± 0.20	0.57 ± 0.28	10.13 ± 4.76	2.35 ± 2.20	0.73 ± 0.13	9	NSR (TSK11, 12, 14), relF0SD (TSK1, 6, 9, 14), SPIR (TSK10), TSR (TSK12)
Total	1.36 ± 0.49	1.67 ± 0.59	13.89 ± 5.04	18.27 ± 13.27	0.75 ± 0.39	6	NSR (TSK7, 12, 14), SPIR (TSK10), TSR (7, 11)
Combination							
Q3	0.52 ± 0.19	0.62 ± 0.21	12.64 ± 4.65	2.48 ± 1.64	0.84 ± 0.16	6	F1SD (TSK9, 11), F2IR (TSK11), jitter (TSK4), TSR (TSK10, 11)
Q4	0.62 ± 0.21	0.77 ± 0.24	16.99 ± 5.83	3.76 ± 2.21	0.71 ± 0.19	5	DDK rate (TSK5), F1IR (TSK11), F2IR (TSK10, 11), TSR (TSK7)
Q5	0.34 ± 0.17	0.45 ± 0.27	7.37 ± 3.75	1.62 ± 2.00	0.86 ± 0.08	8	F2IR (TSK1, 6), F2SD (TSK7, 10), jitter (TSK4), relF0SD (TSK4), relSEOSD (TSK6, 9)
Q6	0.37 ± 0.17	0.48 ± 0.25	8.59 ± 4.00	1.71 ± 1.72	0.81 ± 0.10	2	F1SD (TSK1), SPIR (TSK10)
Total	1.35 ± 0.43	1.61 ± 0.50	13.79 ± 4.42	16.55 ± 10.10	0.79 ± 0.19	3	F1IR (TSK1), F1SD (TSK9), TSR (TSK11)

*MAE* mean absolute error, *RMSE* root mean square error, *EER* estimation error [%], *SSE* sum of squared errors, $R^{2}$ coefficient of determination, *No.* number of selected features

## Discussion

Giladi et al. ([24]), who introduced the FOG–Q, reported that its scores correlated significantly with UPDRS III, which is in line with our findings (in both sessions). This result is to be expected, because several UPDRS items assess freezing, walking and gait as well. This is similar to the results published by Macht et al. ([44]), Nilsson et al. ([51]), and Shine et al. ([65]); we identified that FOG is strongly associated with disease duration. Following this result, we observed FOG–Q scores no longer correlated with LED after the 2-year follow-up assessment. This is an interesting finding pointing out the fact that as the disease progresses, the FOG episodes lose their responsiveness to l-dopa, or the effect of l-dopa is not as easily predictable anymore. Remarkably, two different types of FOG may be distinguished, i. e. FOG during OFF periods may respond to levodopa and deep brain stimulation [35]. “Unresponsive FOG” refers to FOG which is indifferent to changes in dopaminergic medication, whereas ON period freezing occurs only during an ON period and is not present when a patient is OFF [18]. With the disease progression, the unresponsive FOG seems to be more prevalent and other neurotransmitters and pathophysiological mechanisms play more important roles than the dopaminergic deficits [72, 77]. FOG–Q scores significantly correlated with non-motor symptoms assessed by NMSS, which can support findings by Zhang et al. ([79]), who reported association of FOG with the cardiovascular domain of the NMSS. In both sessions, the FOG–Q total score correlated significantly with a level of REM sleep behaviour disorder assessed by RBDSQ. This confirms findings of several studies reporting that increased muscle activity during REM sleep is a comorbid feature of patients with PD who exhibit FOG [74, 76, 79]. Yao et al. ([78]) demonstrated that FOG is related to impaired cognitive functions in PD patients. They found a significant negative correlation between scores of FOG–Q and MMSE. Moreover, in our previous study, we showed that FOG improved the accuracy of prediction of cognitive change in PD [58]. Similarly, we identified negative correlations between FOG–Q and MMSE or ACE-R; however, in our case, none of the correlations were significant. Finally, regarding the BDI, Shine et al. ([65]) studied relationship between FOG and depressive symptoms in PD. They concluded that these symptoms are pertinent and significant predictors of FOG. The association between the FOG and depressive symptoms was reported in other studies [25, 76] and it is also shown by our results, however, only in the follow-up session, probably due to the presence of more depressive symptoms (although not severe enough to diagnose the major depressive episode) at this follow-up visit linked to the progression of the disease (see Table 1).

As for the relations between the acoustic parameters of speech and FOG, it should be mentioned that when employing some kind of significance level adjustment in partial correlations (see Table 5), e. g. using Bonferroni or FDR (false discovery rate) correction, none of the correlations appeared to be significant. Therefore, the results must be considered as exploratory and pilot in nature. Most of the FOG–Q questions, and the Q3–6 total score as well, correlated significantly with the articulation measures, more specifically with the interpercentile range of first formant. Moreover, in some cases, we observed that the standard deviation of the first formant was close to the significance level. Formants are resonances of the oro-naso-pharyngeal tract that are changed mainly by a position of tongue and jaw [3, 31, 32], where the first formant is influenced by a vertical position of these articulatory organs. Therefore the interpercentile range of the first formant is related to the limit positions of the jaw and tongue in the vertical direction, and the standard deviation of this parameter is related to the jaw and tongue tremor (when quantifying sustained phonation) or the speed of articulatory organ position change (when quantifying running speech). All the partial correlations with the formant-based measures (after regressing out the effect of confounding clinical and demographic variables) are negative, i. e. the worst performance in FOG–Q is linked to the worst articulation. This confirms the findings of Ricciardi et al. ([59]), who used a simple one-item articulation analysis using the Dysarthria Profile.

The second most frequent significant measure that appears in Table 5 is based on the speech rate (quantified either by the total speech rate or by the net speech rate). Surprisingly and contrary to the previously published work [59], we identified positive correlations with FOG–Q scores, which means that patients with more severe FOG exhibited a higher speech rate. However, our data shows that with increasing the speech rate, the articulation was less precise (as indirectly measured by the movement of the tongue and jaw). Therefore, we may speculate that a more severe FOG is linked to speech rashes and imprecise (disturbed and less intelligible) speech.

Contrary to the work of A. M. Goberman ([29]), we did not observe any significant correlations with vocal tremor. In general, we did not observe any significant correlations with monopitch and monoloudness. Therefore, based on our results, we may summarize that FOG manifestations are mainly related to improper articulation and disturbed speech rate.

In our recent work, we proved that the acoustic analysis of HD can predict cognitive decline in PD patients [58]. Based on the hypothesis that HD and FOG have some common pathophysiological mechanisms, we assumed that the acoustic features could be also predictors of FOG severity changes. The results in Table 6 support this assumption. For example, a properly selected combination of acoustic measures can predict the change in FOG Q5 score (freezing when initiating the first step) with 7.37 % error, and FOG Q6 (freezing when turning) with an error of 8.59 %. To our best knowledge, this is the first study dealing with acoustic measures of speech as FOG deficit predictors; therefore, the results cannot be compared with the literature.

The exploratory correlation analysis revealed several connections between the acoustic features of HD and the manifestations of FOG. Although the phonatory and some prosodic features were not significant in this analysis, they played some role when combining them with other parameters in order to predict FOG changes. For instance, the lowest error of the FOG Q5 score prediction was 7.37 %, when parameters quantifying monopitch, monoloudness, microperturbations in frequency and tongue/jaw rigidity were combined.

To sum up, although some results are preliminary, it is evident that the acoustic analysis of HD can predict FOG severity change during a 2-year follow-up. This finding may have practical implications since the acoustic recording of speech lasts only several minutes and can thus be easily performed in an outpatient clinic. FOG is associated with falls [53] and thus their severity and the FOG change prediction during the progression of the disease from baseline acoustic speech assessment (performed by a cognitive system) may be of a relevant and clinically important value.

## Conclusions

HD and FOG are both disabling axial symptoms that occur in PD. Although it has been assumed that they have some common pathophysiological mechanisms, a complexity study analysing these mechanisms was missing. As soon as we understand these mechanisms, we can propose new cognitive systems that would assess the difficulties of gait indirectly and predict their change over time. This work is therefore focusing on the description of relationship between HD and FOG employing a quantitative analysis of the HD speech disorders. Moreover, it is the first study to explore a possibility of FOG deficit prediction using acoustic HD analysis.

We extracted a set of the 19 most frequently used acoustic measures quantifying phonation, articulation and prosody in 75 PD patients, who were also assessed by 6 clinical scales, including FOG–Q. Based on a partial correlation analysis, we conclude that FOG is mainly linked to improper articulation (related to limited tongue and jaw movement), disturbed speech rate, and indirectly to speech intelligibility. We have not identified any significant relationship to vocal tremor, monopitch and monoloudness. Finally, using regression models and a properly selected combination of speech/voice features, we proved that acoustic analysis at baseline can predict the change in FOG during a 2-year follow-up with an error in the range of 7.37–16.99 %.

A limitation of this study is the size of the cohort that was analysed. Generally, an acquisition of PD patients is very time-consuming (due to the complex assessment performed by neurologists and clinical psychologists), physically demanding (PD is a movement disorder; therefore, it is usually complicated to get the patients into a hospital), and it is difficult to access a large number of participants (due to prevalence which is estimated to 1.5 % for people aged over 65 years [60]). Moreover, during a couple of years, some patients die or reach an advanced stage of the disease so that they are not able to continue in a 2-year follow-up study. This was also our case. However, to the best of our knowledge, it is still the biggest database that has ever been processed in terms of quantitative FOG and HD relation analysis. To sum up, this study is that of a pilot nature. Although the results are suggesting that the acoustic analysis of HD can be used for the prediction of FOG deficits, our findings should be confirmed by further scientific research that will include bigger and multilingual cohorts.
